# Evaluation of Serum Interleukin-21 and HLA-C1 Polymorphism in Pediatrician Hematopoietic Stem Cell Transplantation for Early Diagnosis of Acute Graft-Versus-Host Disease

**DOI:** 10.18869/acadpub.ibj.21.6.392

**Published:** 2017-11

**Authors:** Nasrin Sehati, Parviz Kokhaei, Ali Motevalizade Ardekani, Raziyeh Tootoonchian, Fatemeh Pak

**Affiliations:** 1 Student Research Committee, Semnan University of Medical Sciences, Semnan, Iran; 2 Cancer Research Center and Department of Immunology, Semnan University of Medical Sciences, Semnan, Iran; 3 National Institute of Genetic Engineering and Biotechnology (NIGEB), Tehran, Iran

**Keywords:** Hematopoietic stem cell transplantation, NK cells, IL-21

## Abstract

**Background:**

Allogenic hematopoietic stem cell transplantation (HSCT) is a strategy used for treatment of different malignant diseases. However, success of allo-HSCT can be hampered by graft-versus-host-disease (GVHD). Natural killer (NK) cells may play an important role in activating antigen presenting cells and subsequent activation of T cells. The main purpose of this study was the evaluation of IL-21, as a blood biomarker, for early detection of acute GVHD (aGVHD) in children after HSCT and also the study of human leukocytes antigen (HLA)-C1 polymorphism, as a targeting ligand for NK cells in these patients.

**Methods:**

Fifty one children receiving HSCT were studied. Blood samples were collected at -8, 7, and 14 days of transplantation. The -8-day samples were analyzed for HLA-C1 polymorphism by PCR-sequence-specific primer technique and pre-transplantation IL-21 assay. To study the serum levels of IL-21, two blood samples were collected on days +7 and +14 and analyzed by ELISA technique.

**Results:**

The results indicated that the incidence of aGVHD in pediatric is associated with a polymorphism of HLA-C1, as alleles HLA-C01:12 (*P*<0.001), HLA-C01:22 (*P*<0.004), and HLA-C01:67 (*P*<0.009). On the other hand, the serum levels of IL-21 in children with aGVHD were decreased after transplantation compared to before transplantation. The serum levels of the IL-21 at 14 days after transplantation had a significant correlation with the occurrence of aGVHD (*P*=0.05).

**Conclusion:**

Based on the findings of this study, there is a significant correlation between HLA-C1 polymorphisms and the serum levels of IL-21 with the incidence of aGVHD.

## INTRODUCTION

Allogenic hematopoietic stem cell tran-splantation (HSCT) is a valuable treatment for a variety of anemias such as thalassemia and malignant hematological diseases such as acute lymphoblastic leukemia and acute myeloid leukemia[1]. The development of graft-versus-host disease (GVHD) limits the success of allogeneic HSCT and is fatal to approximately 14-52% of transplant recipient children[1]. GVHD is the result of immunological attack on target recipient organs or tissues (such as skin, liver, and gut) by donor allogeneic T cells that are transferred along with the allograft. The pathophysiology of acute GVHD (aGVHD) has been described as a three-phase phenomenon. The first phase involves damage to host tissues by inflammation from the preparative chemotherapy and/or radiotherapy regimen. In the second phase, both recipient and donor antigen-presenting cells, as well as inflammatory cytokines trigger the activation of donor-derived T cells, which expand and differentiate into effector cells. In the third (activation) phase, minor histocompatibility antigens play a central role, particularly in the setting of matched sibling transplant. Natural killer (NK) cells have also been shown to have a function in the pathophysiology of aGVHD. NK cells cause tissue damage in phase III (effector phase) of aGVHD by releasing inflammatory cytokines and nitric oxide[1-3].

Preclinical studies have indicated that donor NK cells can suppress aGVHD. The activation of these cells is regulated by a balance between excitatory and inhibitory receptors. Human leukocytes antigen (HLA) class I molecules and killer cell immunoglobulin-like receptors (KIRs) are of pivotal importance for regulating NK[4-7]. The majority of NK cells in peripheral blood expresses at least one inhibitory receptor for self-major histocompatibility complex class I and is functionally competent to recognize and eliminate target cells, which down-regulated the respective major histocompatibility complex class I ligands[8,9].

The role of mismatches KIR and their ligands (HLA-C1/2) has been reported by previous studies. Allogeneic reactions occurred between individuals who have incompatibility between inhibitory KIR–HLA-1. KIR ligand (HLA-C) polymorphism has an important function in NK cell activity and the outcome of HSCT[10-15]. One of the potential factors affecting the success of transplantation is the secretion of cytokines such as IL-2, IL-15, IL-18, IL-12, and IL-21 from immune cells[14]. IL-21 is a cytokine with potent regulatory effects on cells of the immune system, including NK cells, Th1, Th17, and cytotoxic T cells that can destroy virally infected or cancerous cells. This cytokine induces the division/proliferation of its target cells. The IL-21 receptor is expressed on the surface of T, B, and NK cells. To determine the role of IL-21 in GVHD, anti-IL-21 antibody was given to recipients of CD25−CD4+ or CD4+ and CD8+ T-effectors in mice. IL-21 neutralization attenuated GVHD-related weight loss and resulted in prolonged survival[28]. IL-21 signaling has a key role in the deaths from aGVHD in a mice model. Furthermore, the morbidity and mortality of GVHD were significantly reduced after bone marrow transplantation in IL-21R−/− mice relative to those from wild-type mice[29-30]. Immunological effects of IL-21 on T, B, and NK cells have already been reported, but the role of IL-21 in GVHD in human remains obscure. The main objective of the present study was to examine the HLA-C1 polymorphism, as a targeting ligand for NK cells, and to evaluate the serum level of IL-21 in HSCT recipients, as one of the most important cytokines in the activation of immune cells such as NK, B, and T cells.

## MATERIALS AND METHODS

### Patient selection

In total, 51 children who received HSCT in Shariati Hospital in Tehran (Iran) were studied. All children were diagnosed with different types of anemia, immune deficiency, and childhood cancer. The patients were evaluated for the underlying disease, the source of hematopoietic cells (peripheral blood, bone marrow, and umbilical cord), and also gender (Table 1). The mean follow-up time of the patients was 100 days. Patients with veno-oclusive disease, idiopathic pneumonia syndrome, and those with symptoms of septic shock were excluded. After obtaining informed consents, the study was approved by local ethical committee at Semnan University of Medical Sciences (Semnan, Iran).

**Table 1:** Patients and transplant characteristics

Characteristic	N
Gender	
Male	35
Female	16
Disease	
Thalassemia	24
Fanconi anemia	5
AML	7
ALL	4
Other (LAD=1, CMML=1, CGD=3, Aplastic Anemia=3, Sickle cell anemia=1, DBA=1, MDS=1)	
Type of graft	
Allo B.M.	25
Allo P.B.	20
Cord blood	6
Type of reaction	
Acute GVHD	14
Non-GVHD	37

Allo B.M., allogenic bone marrow; Allo P.B., allogenic peripheral blood; AML, acute myeloid leukemia; N, number

### Human leukocytes antigen genotyping

HLA typing was carried out for the patients, sibling bone marrow donors, and parents. HLA-A, HLA-B, and HLA-DR were performed using the PCR-sequence-specific primer (SSP) method[17]. Donor selection criteria required full matching for defined HLA alleles. Blood samples were collected eight days Allo B.M., allogenic bone marrow; Allo P.B., allogenic peripheral blood; AML, acute myeloid leukemia; N, number before transplantation. HLA-C1 polymorphisms were determined by low-resolution DNA-based typing using PCR-SSP (Olerup, Sweden).

### Detection of serum IL-21

Serum samples at eight days before transplantation (-8), as well as 7 (+7) and 14 (+14) days after HSCT were collected for assessments and analysis. The evaluation of the serum level of IL-21 was carried out using ELISA method.

### Statistical analysis

aGVHD was defined as development of grade II to IV during first 100 days post transplantation. The patients were divided into two groups: children who had endured transplant and children with aGVHD. The polymorphisms linked to incidence and the severity of aGVHD in children in both groups were studied. Statistical significance between the two groups was calculated using SPSS software version 16. The nominal significance level was set to 0.05.

## RESULTS

Of 51 children, 14 were diagnosed with aGVHD and 37 cases had no symptoms of aGVHD. The incidence of aGVHD was estimated to be 27%. In this study, HLA-C1 polymorphisms, as the KIR ligand, were performed. Based on statistical analysis, HLA-C01:12 (*P*=0.001), HLA-C01:22 (*P*=0.004), and HLA-C01:67 (*P*=0.009) were identified as susceptible alleles in the aGVHD disease. However, the HLA-C01:26 (*P*= 0.225), HLA-C01:32 01:40 (*P*=0.414), and HLA-C01:84 (*P*=0.543) in those who did not develop aGVHD were identified as protective alleles.

According to Table 2, most of the patients (positive aGVHD) had HLA-C01:12, HLA-C01:22, and HLA-C01:67 alleles, respectively. Also, in the control group (negative aGVHD), HLA-C01:26 allele frequency was high.

**Table 2:** HLA frequency in pediatric hematopoietic stem cell transplant recipients

HLA type	Count (%)	Disease	

		Positive aGVHD	Negative aGaVHD
HLA_C01_84	Count	0.0	2
	within factor	00.0	100
	within disease	00.0	5.5
	of Total	00.0	4.1
HLA_C01_32 01:40	Count	0.0	7
	within factor	0.0	100.0
	within disease	0.0	19.4
	of Total	0.0	14.0
HLA_ C_67	Count	1	0.0
	within factor	100.0	0.0
	within disease	7.1	0.0
	of Total	2.0	0.0
HLA_C01_22	Count	3	1
	within factor	75.0	25.0
	within disease	21.4	7.1
	of Total	6.0	2.0
HLA_C01_12	Count	10	0.0
	within factor	100.0	0.0
	within disease	66.7	0.0
	of Total	20.4	0.0
HLA_C01_26	Count	0.0	26
	within factor	0.0	100.0
	within disease	0.0	76.5
	of Total	0.0	53.1
Total	Count	14	36
	within factor	30.6	69.4
	within disease	100.0	100.0
	of Total	30.6	69.4

HLA, human leukocytes antigen; aGVDH, acute graft-versus-host-disease

Table 3 shows the relationship between the risk of aGVHD in patients and HLA-C01. Patients with HLA-C01:22, HLA-C01:12, and HLA-C01:67 poly-morphisms had the higher risk of disease than those who did not have signs of aGVHD.

**Table 3:** Odds ratio and relative risk between HLA and aGVHD

HLA	Odds ratio for factor value	Relative risk	*P* value
HLA_C01:84	0. 881	1.594	0.543
HLA_C01:32 01:40	0.438	0.531	0.414
HLA_C01:67	1.094	1.062	0.009*
HLA_C01:22	4.375	2.125	0.004*
HLA_C01:12	24.062	2.922	0.001*
HLA_C01:26	0.088	0.123	0.225

* statistically significant; HLA, human leukocytes antigen

**Fig. 1:**
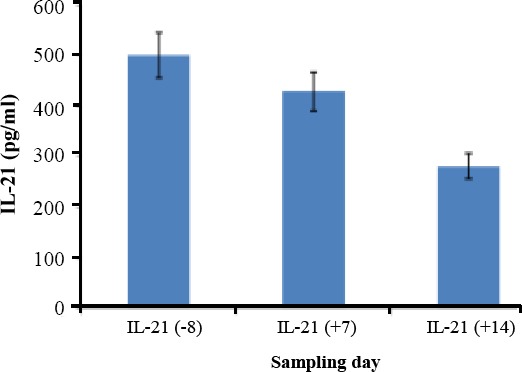
Evaluation of Serum IL-21 before and after HSCT.

The results of statistical analysis showed a significant relationship between the serum level of IL-21 and the gender of patients. The serum level of IL-21 in females was significantly higher than male children (Table 4). The result also depicted that the levels of IL-21 at 14 days after transplantation were reduced significantly at *P*<0.5 (Fig. 1).

**Table 4:** The relationship between the serum levels of IL-21 with the gender of patients

Cytokine	Gender	N	Mean (Pg/ml)	*P* value
IL-21(-8)	Male	33	406.85	
	Female	18	621.88	0.005
IL-21(+7)	Male	33	375.21	
	Female	18	534.81	0.082
IL-21(+14)	Male	33	213.94	
	Female	18	410.74	0.013

N, number

**Fig. 2:**
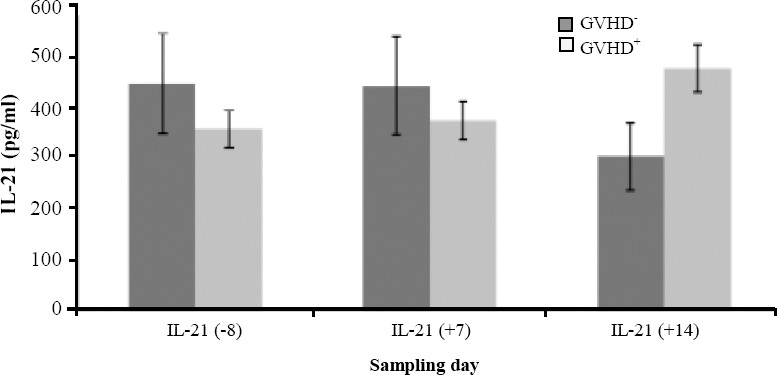
Serum IL-21 in patients with aGVHD and patients who did not develop signs of aGVHD.

The level of serum IL-21 in the aGVHD-positive group was increased after HSCT and reached maximum at 14 days after transplantation, while downward trend was observed in the group that was negative for aGVHD (Fig. 2). Table 5 shows that the serum level of IL-21 at 14 days after transplantation has a meaningful relationship with the incidence of aGVHD (Table 5).

**Table 5:** The relationship between IL-21 and the incidence of aGVHD

Cytokine	GVHD	N	Mean (pg/ml)	*P* value
IL-21(-8)	Positive	14	371.35±91
	Negative	37	462.10±101	0.60
IL-21(+7)	Positive	14	387.14±79
	Negative	37	458.30±81	0.62
IL-21(+14)	Positive	14	493.35±32	
	Negative	37	314.80±59	0.05*

* statistically significant; GVDH, graft-versus-host-disease

## DISCUSSION

Despite improvements in our understanding of transplant immunology, both aGVHD and chronic GVHD remain a clinical challenge and a major cause of morbidity and mortality for HSCT recipients. Although testing for HLA-B, HLA-A, and HLA-DR polymorphisms before transplantation for the selection of the best donor reduces the risk of GVHD to 14-52%, additional studies are needed to reduce the incidence of GVHD after HSCT[2].

New studies on the role of immune cells in the pathogenesis of GVHD and studying the function of innate immune cells, especially NK cells, are of interest[4]. Besides, the NK cells in the pathophysiology of aGVHD, some studies have shown the role of alloreactive NK cells in outcome of transplantation in animal models[12-16]. Cicconeet *et al*.[16], followed by Döhring[12] in 1996, reported that NK cells are able to kill allogeneic cells expressing HLA class-1 alleles but are not recognized by the inhibitory NK cells of recipient. In the same year, Ruggeri and co-workers[14] showed that the signal transmission by KIRs (regulatory surface molecules found on NK cells) has important function in NK cell activity and HSCT outcome in acute myeloid leukemia patients[12-16]. These receptors interact with the certain motifs of HLA-1. La Nasa and colleagues[17] studied KIR and HLA-C genotype in a homogeneous group of 45 thalassemia patients undergoing bone marrow transplantation. Their results showed that heterogeneity of this ligand takes an important part in the incidence of aGVHD.

Nguyen *et al*.[18] and McQueen *et al*. [19] and showed that in the early phases of aGVHD, the frequency of NK cells with markers of KIR2DL2/2 and KIR2DS2, which bind to HLA-C1, is higher than NK cells with KIR2S1 and KIR2DL1 receptors binding to HLA-C2. It seems that in some cases, HLA-KIR incompatibility leads to beneficial allogeneic reactions. For the first time, Ruggeri *et* *al*.[15] showed these effects in his study. They removed T cells and then chose the best donor according to HLA incompatibility test. The result indicated that overall survival was increased, and aGVHD, rejection, and recurrence were reduced in

HLA, human leukocytes antigen; aGVDH, acute graft-versus-host-disease recipient mice. However, a previous study involving heterogeneous recipients from unrelated donors did not show beneficial effect on aGVHD, rejection, and recurrence. Therefore, it can be concluded that KIR-HLA incompatibility does not offer any advantages. Bornhäuser *et al*.[21] failed to confirm the beneficial effects of KIR-HLA in compatibility. However, Hsu *et al*.[22], in a HLA-KIR compatibility study indicated that patients with myelodysplastic syndrome who received HSCT showed beneficial effect. In the present study, HLA-C1 polymorphism determination, as the KIR ligand, were performed by PCR-SSP method. We obtained the following results: Based on *P* value, OR (odds ratio), and RR (relative risk) inTables 2 and 3, HLA-C01:12 (frequency 4/20%, *P*=0.001, OR: 24.6, and RR: 2.922) and HLA-C01:22 (frequency 4/21%, *P*=0.004, OR: 4.37, and RR: 2.25) were identified as susceptible alleles in the aGVHD disease. However, the HLA-C01:26, HLA-C01:32 01:40, and HLA-C01:84, with the frequencies of 5.76%, 4.19%, and 5.5%, respectively, in those who did not develop aGVHD were identified as protective alleles.

Overall, the present study demonstrated that HLA-C1 polymorphism is effective in aGVHD occurrence after HSCT in children. The results also indicated that KIR-HLA compatibility is most probably important to the incidence of aGVHD after pediatrician HSCT. The human killer cell Ig-like receptor (KIR) locus comprises two groups of KIR haplotypes, termed A and B. These are present in all human populations but with different relative frequencies, suggesting they have various functional properties that underlie their balancing selection.

We studied the genomic organization of the alleles of HLA-C. Because every HLA-C allotype functions as a ligand for KIR, the interactions between KIR and HLA-C dominate the HLA class I-mediated regulation of human NK cells. As noted before, in addition to immune cells, cytokines have a strong role in the pathophysiology of aGVHD. IL-21 is produced by TH-17 cells and causes the development of B cells, plasma cells, NK, and T cells[23,24]. It also causes an increase in the cytotoxic activity of NK and T CD8+ cells[25] and has antitumor effects as well[26].

In 2009, Bucher *et al*.[27], in an *in vivo* study on mouse models, revealed that the inhibition of IL-21 receptor signal transmission reduces the incidence of aGVHD along with the number of TH-1 cells in intestinal mucosa. Following that study, Hippen *et al*.[28] demonstrated similar results with the use of anti-IL-21 antibody. Meguro and colleagues[29] reported that the absence of IL-21 signal transmission reduced the effects of graft-versus-leukemia.

Hanash *et al*.[30] found the same results in the same year. In 2013, Wu *et al*.[31] examined the effect of IL-21 on GVHD in mouse models and showed that the increase of B-cell proliferation caused the development of GVHD by IL-21. In the present study, the results showed that the serum level of IL-21 in children is reduced only after transplantation (Fig. 1). The results of statistical analysis displayed a significant relationship between the serum level of IL-21 and the gender of patients. The level of IL-21 in female was more than male (Table 4). Immune responses differ between the genders. In addition to behavioral, genetic, and hormonal factors, differences in the abundance and activation of various types of immune cells could explain some of the observed sexual dimorphisms. The relative proportions of certain immune cell populations vary between men and women. Women had lower monocyte counts but a higher percentage of T lymphocytes within the total lymphocyte population, and the executive cells in aGVHD were T cells.

The current study compares the serum level of IL-21 in two groups, children who developed aGVHD after the transplantation and those who did not. The results indicated that the serum level of IL-21 in the group suffering from aGVHD in comparison to the group in which children did not develop the disease has an upward trend (*P*=0.05). These findings are consistent with the results of previous studies in mouse models[12,14,31].

Based on the findings of the present study (Table 5), after seven days of HSCT, the serum level of IL-21 was not meaningfully different from the level of this cytokine before HSCT. However, the level of IL-21 at 14 days after HSCT has been shown a meaningful relationship with the incidence of aGVHD (*P*=0.05). It seems that in two weeks after transplantation, during the engraftment of cells in the recipient, IL-21 secretion has possibly been increased by the cells. It is estimated that during the following days, the secretion of this cytokine increases substantially. Moreover, the findings of the present study may suggest an important role for IL-21 in severity of aGVHD in children after HSCT. In addition, the evaluation of IL-21 can be useful to predict the incidence of aGVHD in pediatric patients. Further studies are necessary to elucidate the role of cytokine IL-21 in the severity of inflammatory and immune responses in children suffering from aGVHD. Based on our findings, there is a significant correlation between serum levels of IL-21 and HLA-C1 polymorphisms with the incidence of aGVHD, and this may be useful in predication of aGVHD risk in children after HSCT.
